# Correlation between IL-6 and S-100B blood levels and outcome of post-cardiac arrest syndrome and influence of therapeutic hypothermia on these mediator blood levels

**DOI:** 10.1186/cc10884

**Published:** 2012-03-20

**Authors:** K Shinozaki, S Oda, T Sadahiro, M Nakamura, E Watanabe, R Abe, T Nakada, Y Morita, K Nakanishi, N Kitamura, H Hirasawa

**Affiliations:** 1 Chiba Aoba Municipal Hospital, Chiba City, Japan; 2 Chiba University, Chiba City, Japan; 3 Narita Red Cross Hospital, Narita City, Japan; 4 Kimitsu Chuo Hospital, Kisarazu City, Japan

## Introduction

To elucidate the significance of IL-6, S-100B and NSE in pathophysiology of post-cardiac arrest syndrome (PCAS), blood levels of those mediators sampled within the first 24 hours after cardiac arrest (CA) were compared between groups classified according to survival and neurological outcomes. Furthermore, influence of stability of core temperature with therapeutic hypothermia (TH) on these mediator blood levels was also investigated.

## Methods

Nontraumatic out-of-hospital CA patients were included. Blood was sampled on admission, at 6 hours and 24 hours after CA, respectively. Then, patients that died within 24 hours after CA were excluded. Patients were classified into nonsurvivors (died within 28 days) and survivors (survived for 28 days or longer), and classified into poor neurological outcome (CPC 3 to 5) and favorable neurological outcome (CPC 1 to 2), respectively. Factors significantly correlated with survival and neurological outcomes were investigated by comparing baseline characteristics and mediator blood levels. Patients receiving TH were also included into subgroup analysis. If the core temperature was maintained at 33 ± 1°C for more than 18 hours within the first 24 hours, the patient was classified into maintained, and if not into not-maintained, and mediator blood levels were compared between the subgroups.

## Results

One-hundred and four patients survived more than 24 hours out of all 1,026 patients analyzed. Mean IL-6, S-100B and NSE levels in nonsurvivors (*n *= 51) were significantly higher than those in survivors (*n *= 53) at all timepoints (*P *< 0.01). Those in poor neurological outcome (*n *= 74) were significantly higher than those in favorable neurological outcome (*n *= 29) at all timepoints (*P *< 0.01). From the results of ROC analysis and multivariate analysis, IL-6 >240 pg/ml at 6 hours and S-100B >0.37 ng/ml on admission were chosen as an independent predictors of nonsurvival, and S-100B >0.07 ng/ml at 24 hours was chosen as that of poor neurological outcome. Subgroup analysis of 56 patients showed that mean levels of IL-6 at 6 hours, S-100B at 6 hours and S-100B at 24 hours in the maintained (*n *= 29) group were significantly lower than those in the not-maintained group (*n *= 27) (*P *< 0.05).

## Conclusion

IL-6 and S-100B levels within 24 hours after CA, but not NSE, are related to survival and neurological outcome. IL-6 and S-100B are considered to be important mediators for the pathophysiology of PCAS and TH may influence blood levels of these mediators.

